# *Helicobacter pylori* Infection and Insulin Resistance in Diabetic and Nondiabetic Population

**DOI:** 10.1155/2014/391250

**Published:** 2014-10-23

**Authors:** Jamshid Vafaeimanesh, Mahmoud Parham, Mohammadreza Seyyedmajidi, Mohammad Bagherzadeh

**Affiliations:** ^1^Clinical Research Development Center, Department of Internal Medicine, Qom University of Medical Sciences, Qom, Iran; ^2^Golestan Research Center of Gastroenterology and Hepatology (GRCGH), Golestan University of Medical Sciences, Gorgan, Iran

## Abstract

*Helicobacter pylori* (*HP*) is a common worldwide infection with known gastrointestinal and nongastrointestinal complications. One of the gastrointestinal side effects posed for this organism is its role in diabetes and increased insulin resistance. The aim of this study was to evaluate the association between *HP* and insulin resistance in type 2 diabetic patients and nondiabetics. This cross-sectional study was carried out from May to December 2013 on 211 diabetic patients referred to diabetes clinic of Shahid Beheshti Hospital of Qom and 218 patients without diabetes. *HP* was evaluated using serology method and insulin resistance was calculated using HOMA-IR. The prevalence of *H. pylori* infection was 55.8% and 44.2% in diabetics and nondiabetics (*P* = 0.001). The study population was divided into two *HP* positive and negative groups. Among nondiabetics, insulin resistance degree was 3.01 ± 2.12 and 2.74 ± 2.18 in *HP*+ and *HP*− patients, respectively (*P* = 0.704). Oppositely, insulin resistance was significantly higher in diabetic *HP*+ patients rather than seronegative ones (4.484 ± 2.781 versus 3.160 ± 2.327, *P* = 0.013). In diabetic patients, in addition to higher prevalence of *HP*, it causes a higher degree of insulin resistance.

## 1. Introduction

The most common infection in the world, especially in developing countries, is* Helicobacter pylori* (*HP*) infection which is an etiological factor for developing peptic ulcer, gastric cancer, and acute polymorphonuclear infiltration in the gastric mucosa [1, 2]. The mononuclear infiltration is characterized by the local production and systemic diffusion of proinflammatory cytokines affecting remote tissues and organic systems [3, 4]. This systemic inflammation causes some extragastrointestinal side effects of* HP* including ischemic heart disease [1], sideropenic anemia [5], idiopathic thrombocytopenic purpura [6], neurologic diseases [7], and hepatobiliary diseases [8].

It has been shown that* HP* infection plays a role in some endocrine disorders, such as autoimmune thyroid diseases, diabetes, and primary hyperparathyroidism and may have a high prevalence among patients with diabetes [9, 10]. The association between* HP* and diabetes was first explored in Simon et al.'s study [11]. Recently, a meta-analysis [12] showed* HP* infection was increased to 1.33 among patients with diabetes. In addition, some studies have shown an increased incidence of diabetes among people with* HP* infection so that the first report that* HP* infection increased incidence of diabetes was in a study by Jeon et al. [13] using a prospective cohort of 782 Latino individuals >60 years of age.

Etiopathogenesis of* HP* infection in diabetic patients has not been defined clearly. However, this hypothesis is now proposed that* HP* infection is more prevalent among people with diabetes but it is not clear whether diabetics have more susceptibility to this infection or* HP* infection increases the susceptibility to diabetes. One of the hypotheses about* HP* infection as a risk factor for diabetes is increased insulin resistance in these patients.

As insulin resistance can develop in the presence of inflammation or as a result of alterations in counter regulatory hormones that affect insulin,* HP* may thus promote insulin resistance by inducing chronic inflammation and affecting insulin-regulating gastrointestinal hormones [14, 15]. The first direct evidence for an association between chronic* HP* infection and insulin resistance rose from Aydemir et al.'s study [15] showing higher homeostatic model assessment-estimated insulin resistance (HOMA-IR) scores in* HP* positive (*HP*+) individuals. In contrast, some studies have not shown this association [16].

Regarding these conflicting findings and the fact that proving a causal role between* HP* infection and insulin resistance increase has important role in controlling the important and common diseases such as diabetes and nonalcoholic fatty liver, we aimed to evaluate the association between* HP* infection and insulin resistance in type 2 diabetic patients and nondiabetics.

## 2. Materials and Methods

### 2.1. Patients

This cross-sectional study was carried out from May to December 2013 on 211 type 2 diabetic patients referred to diabetes clinic of Shahid Beheshti Hospital of Qom and 218 nondiabetic patients. Type 2 diabetic patients with diabetes duration of >5 years and receiving oral hypoglycemic drugs participated in this study as case group and the control group was selected from attendants with the patient referring to the endocrinology clinic. Diabetes diagnosis was based on American Diabetes Association's criteria (fasting plasma glucose diagnostic of ≥126 mg/dL, 2-hour plasma glucose value of ≥200 mg/dL, and HbA1c of ≥6.5%) [17].

In case of receiving insulin, pregnancy, smoking, history of* HP* treatment (proton-pump inhibitor, H2 blocker, and bismuth) or receiving antibiotics in the previous 6 months, surgery on upper GI tract, gastric cancer, and using nonsteroidal anti-inflammatory drugs, patients were excluded from the study. Then, the presence of* HP* infection patients was evaluated. After 8-hour fasting overnight, blood samples were taken at 4°C and, right after sampling, the serum was centrifuged at 2000 rpm for 15 minutes. Anti-*HP* IgG antibody was measured by ELISA kit, made by Padtan Elm Co, Iran. In case of serum titer above 30 AU/mL, it was considered positive. Serum insulin was measured using ELISA kit, DiaMetra Co, Italy. We calculated and compared homeostasis model assessment of insulin resistance (HOMA-IR) in this study multiplying the fasting glucose value (mg/dL) by serum insulin value in each person and then dividing it by 405.

### 2.2. Statistical Analysis

Data analysis was performed by SPSS version 16.0 using descriptive statistics, mean, standard deviation, percentage, and frequency. The analysis was performed by *t*-test, Chi-square test, and Fisher's exact test.

### 2.3. Ethics

All individuals signed informed consent prior to their enrolment in the study. Also, the study was planned according to the ethical guidelines following the Declaration of Helsinki and Ethics Committee of Qom University of Medical Sciences approved it.

## 3. Results

In this study, 211 diabetic and 218 nondiabetic subjects were studied. Female/male ratio in patients with diabetes and nondiabetic patients was 135/76 and 109/109, respectively. In patients with diabetes, the prevalence of* HP* infection was 55.8% while it was 44.2% in nondiabetics. The study population was divided into two* HP* positive and negative groups. As it is shown in Table 1, patients with diabetes and nondiabetic subjects were not significantly different in terms of gender, age, body mass index, and physical activity level.

Among nondiabetic subjects, HOMA-IR score was 3.01 ± 2.12 and 2.74 ± 2.18 in* HP*+ and* HP*− patients, respectively. Although insulin resistance was higher in* HP*+ individuals, this difference was not statistically significant (*P* = 0.704). Oppositely, insulin resistance was significantly higher in* HP*+ diabetic patients rather than seronegative ones (4.48 ± 2.78 versus 3.16 ± 2.32, *P* = 0.013).

As shown in Table 2, although the blood sugar level in* HP*+ diabetic patients was higher than* HP*− subjects, the difference was not statistically significant (*P* = 0.468). Evaluation of lipid profile showed that the only significant difference between both groups was lower HDL among* HP*+ diabetic patients (60.7 ± 26.7 versus 69.2 ± 29.2, *P* = 0.037) (Table 2).

## 4. Discussion

In this study the rate of* HP* seropositivity among diabetic and nondiabetic patients was patients and nondiabetics and the difference between the groups was statistically significant (*P* = 0.001) [10]. Another seroprevalence study in United Arab Emirates showed that positive antibody titer for* HP* infection (IgA > 250) in diabetics was 63.3% compared to nondiabetics 48.1%; similarly, according to IgG antibody titer (IgG > 300),* HP* infection was determined in diabetic patients at a rate of 76.7% compared to an infection rate of 64.8% in nondiabetics [18]. Candelli and colleagues found that the prevalence of* HP* infection was higher in diabetics (24%) than in controls of similar age, gender, and socioeconomical status after three years of follow-up and the reinfection rate was higher in diabetic patients [19].

In contrast to these studies, other researchers did not report this association [20–22]. It is not clear whether diabetics have more susceptibility to this infection or* HP* infection increases the susceptibility to diabetes. One of the hypotheses about* HP* infection as a risk factor for diabetes is increased insulin resistance in these patients. In some studies, people with diabetes are considered at risk of* HP* infection and it is suggested that autonomic neuropathy and poor glycemic control might have a significant role in this field [10].

An association between* HP* infection and changes in gastric motility, acid secretion, and diabetes-induced impairment of cellular and humoral immunity has been reported in several studies [11–13, 23]. Also, altered glucose metabolism may produce chemical changes in the gastric mucosa due to altered glucose metabolism [24] and individuals with diabetes are more frequently exposed to pathogens than their healthy counterparts as they regularly attend hospital settings [25].

In contrast, some investigators believe that* HP* infection is a more favorable condition for developing diabetes. For example, it is commonly believed that the chronic inflammation induced by* HP* infection is strongly associated to the pathogenesis of diabetes, which is linked to a general activation of the innate immune system, and a chronic, cytokine-mediated state of low-grade inflammation. Proinflammatory cytokines affect many tissues which cause recognizable features of diabetes [26].

Inflammation of the adipose tissue is considered a key factor in the pathogenesis of insulin resistance and *β*-cell potential autoinflammation impairs insulin secretion in diabetes.* HP*-induced gastritis can affect the secretion of gastric-related hormones such as leptin and ghrelin, as well as gastrin and somatostatin, which may influence a predisposition to diabetes [27, 28].

One of the hypotheses about* HP* infection as a risk factor for diabetes is increased insulin resistance in these patients. One of the first studies in this field was in Aydemir et al.'s study in 2005 on 63* HP*+ and 27* HP*− patients. Age, gender, and BMI were not different between both groups. HOMA-IR level was 1.73 ± 1.1 in* HP*− group, whereas it was 2.56 ± 1.54 in* HP*+ group. This study addressed the association between* HP* and insulin resistance, although the sample size was small [15].

In 2009 Gunji and colleagues [29] have studied 1107 nondiabetic Japanese patients and found that among those with higher insulin resistance score (HOMA-IR ≥ 2.5), the prevalence of the* HP* was higher (39.4 versus 28.7%, *P* = 0.027). Although people with higher insulin resistance were fewer (99 cases versus 1008), a recent systematic review for the association between* HP* infection and quantitative indexes of insulin resistance showed a positive association between* HP* infection and insulin resistance, independent of several confounders [30].

On the contrary, opposite studies exist too. For example, Gillum stated that there is no consistent association between* HP* infection and diabetic prevalence or variables of the insulin resistance syndrome in American men 40–74 years of age [31]. Also, Malamug and colleagues' study in 2014 was in accordance with that study [16].

In our study insulin resistance was significantly higher in diabetic patients with* HP* infection (4.484 ± 2.781 versus 3.160 ± 2.327, *P* = 0.013). In contrast, although in* HP*+ nondiabetic patients insulin resistance was higher than seronegative individuals (3.01 ± 2.12 versus 2.74 ± 2.18), it was not statistically significant (*P* = 0.704). These findings suggest the hypothesis that* HP* infection does not increase the risk of diabetes in nondiabetic population. But in diabetics,* HP* is more prevalent and also* HP*+ diabetics have a greater HOMA-IR score and need higher levels of insulin for glycemic control.

This study warrants further studies aimed at clarifying which* HP* strain is involved in insulin resistance, whether or not inflammation is involved, and whether inflammatory cytokines are present (or genetics for proinflammatory haplotypes). Also will cure of the pathogen alter the insulin resistance?

However, further studies are needed to investigate the association between* HP* and insulin resistance because understanding the causal role of this organism in insulin resistance is important for controlling the important and common diseases such as diabetes and nonalcoholic fatty liver.

## 5. Conclusion

*HP* infection does not significantly increase insulin resistance in nondiabetic individuals but in diabetic patients; in addition to higher prevalence of* HP*, it causes a higher degree of insulin resistance and needs higher levels of insulin for the same control similar to seropositive subjects.

## Figures and Tables

**Table 1:** The characteristics of the patients with respect to seropositivity for *HP*.

Parameter		*HP*– *N* (%)	*HP*+ *N* (%)	*P* value
Gender (female/male)	Nondiabetic	57 (52.3%)/51 (46.8%)	52 (47.7%)/58 (53.2%)	0.489
	Diabetic	47 (65.3%)/25 (34.7%)	88 (63.3%)/51 (36.7%)	0.778

Insulin resistance risk factors				
Age (year)	Nondiabetic	39.18 ± 12.44	39.88 ± 12.50	0.669
	Diabetic	51.5 ± 8.3	53.5 ± 9.0	0.128
BMI	Nondiabetic	26.25 ± 5.04	26.24 ± 4.33	0.871
	Diabetic	29.4 ± 4.9	28.8 ± 4.8	0.427

Exercise degree				
>150 min per week	Nondiabetic	4 (3.7)	2 (1.8)	0.299
	Diabetic	2 (2.9%)	11 (7.9%)	0.478
30–150 min per week	Nondiabetic	14 (13.0)	7 (6.4)
	Diabetic	4 (5.7%)	10 (7.2%)
<30 min per week	Nondiabetic	27 (25.0)	37 (33.6)
	Diabetic	15 (21.4%)	26 (18.7%)
No	Nondiabetic	63 (58.3)	64 (58.2)
	Diabetic	51 (72.9%)	92 (66.2%)

**Table 2:** Insulin resistance, glycemic control, and medication type in patients with respect to seropositivity for *HP*.

Parameter		*HP*−	*HP*+	*P* value
Serum insulin (*μ*IU/mL)	Nondiabetic	11.65 ± 4.88	12.71 ± 4.64	0.708
	Diabetic	6.97 ± 2.64	10.12 ± 4.72	0.002
HOMA-IR score	Nondiabetic	2.74 ± 2.18	3.01 ± 2.12	0.704
	Diabetic	3.16 ± 2.32	4.48 ± 2.78	0.013
FBS (mg/dL)	Nondiabetic	92.75 ± 11.70	92.58 ± 14.92	0.915
	Diabetic	173.43 ± 61.32	180.12 ± 64.27	0.468

Lipid profile				
HDL (mg/dL)	Nondiabetic	54.23 ± 19.79	56.55 ± 20.05	0.390
	Diabetic	69.2 ± 29.2	60.7 ± 26.7	0.037
LDL (mg/dL)	Nondiabetic	83.26 ± 29.75	85.96 ± 32.67	0.525
	Diabetic	107.1 ± 43.2	116.0 ± 51.7	0.212
TG (mg/dL)	Nondiabetic	142.63 ± 112.27	140.81 ± 95.20	0.897
	Diabetic	224.2 ± 100.2	229.3 ± 114.6	0.747
Cholesterol (mg/dL)	Nondiabetic	161.16 ± 42.68	157.50 ± 38.86	0.509
	Diabetic	205.1 ± 63.2	207.3 ± 67.4	0.8
